# Effect of Copper on the Mitochondrial Carnitine/Acylcarnitine Carrier Via Interaction with Cys136 and Cys155. Possible Implications in Pathophysiology

**DOI:** 10.3390/molecules25040820

**Published:** 2020-02-13

**Authors:** Nicola Giangregorio, Annamaria Tonazzi, Lara Console, Mario Prejanò, Tiziana Marino, Nino Russo, Cesare Indiveri

**Affiliations:** 1 CNR Institute of Biomembranes, Bioenergetics and Molecular Biotechnologies (IBIOM) via Amendola 122/O, 70126 Bari, Italy; n.giangregorio@ibiom.cnr.it (N.G.); a.tonazzi@ibiom.cnr.it (A.T.); 2 Department DiBEST (Biologia, Ecologia, Scienze della Terra) Unit of Biochemistry and Molecular Biotechnology, University of Calabria, Via Bucci 4C, 87036 Arcavacata di Rende, Italy; lara.console@unical.it; 3 Department CTC (Chemistry and Chemical Technology) University of Calabria, Via Bucci 14C, 87036 Arcavacata di Rende, Italy; mario.prejano@unical.it (M.P.); tiziana.marino65@unical.it (T.M.); nino.russo@unical.it (N.R.)

**Keywords:** carnitine, copper, membrane transport, toxicology, computational chemistry

## Abstract

The effect of copper on the mitochondrial carnitine/acylcarnitine carrier (CAC) was studied. Transport function was assayed as [^3^H]carnitine/carnitine antiport in proteoliposomes reconstituted with the native protein extracted from rat liver mitochondria or with the recombinant CAC over-expressed in *E. coli*. Cu^2+^ (as well as Cu^+^) strongly inhibited the native transporter. The inhibition was reversed by GSH (reduced glutathione) or by DTE (dithioerythritol). Dose-response analysis of the inhibition of the native protein was performed from which an IC_50_ of 1.6 µM for Cu^2+^ was derived. The mechanism of inhibition was studied by using the recombinant WT or Cys site-directed mutants of CAC. From the dose-response curve of the effect of Cu^2+^ on the recombinant protein, an IC_50_ of 0.28 µM was derived. Inhibition kinetics revealed a non-competitive type of inhibition by Cu^2+^. However, a substrate protection experiment indicated that the interaction of Cu^2+^ with the protein occurred in the vicinity of the substrate-binding site. Dose-response analysis on Cys mutants led to much higher IC_50_ values for the mutants C136S or C155S. The highest value was obtained for the C136/155S double mutant, indicating the involvement of both Cys residues in the interaction with Cu^2+^. Computational analysis performed on the WT CAC and on Cys mutants showed a pattern of the binding energy mostly overlapping the binding affinity derived from the dose-response analysis. All the data concur with bridging of Cu^2+^ with the two Cys residues, which blocks the conformational changes required for transport cycle.

## 1. Introduction

Copper is an essential cofactor of many cellular molecular systems. Thus, it needs to be absorbed with diet and distributed to the various regions of the body for reaching the final destination and assembly into proteins. The traffic of copper requires several proteins for both crossing the cell membranes and for being shuttled [1]. A very important site of destination for copper is the mitochondria, in which it is a cofactor of enzymes, which include proteins belonging to the respiratory chain complexes [2,3]. Thus, a transport system for copper is needed for crossing the inner mitochondrial membrane and for being assembled into the protein complexes. It was demonstrated that, in yeast, a member of the mitochondrial carrier family is responsible for copper transport from the cytosol to the mitochondrial matrix, namely the phosphate carrier (Pic2) [4]. More recently, similar results were described for mammalian mitochondria. It was shown that the isoform 2 of the phosphate carrier (PIC, SLC25A3) is responsible for copper transport into the matrix [5]. In parallel with the copper traffic within the cell, and especially in mitochondria [2], some off-target interactions with proteins that are not directly involved in copper assembly are expected. In the intermembrane space (IMS), copper may give side interactions with other molecular components, which possess amino acid groups reactive towards copper [6]. In this framework, it was previously demonstrated that the ornithine/citrulline transporter (SLC25A15) can be targeted by copper. The interaction causes alterations of the transport functions that result in inactivation of the physiological transport activity or in the conversion of the transporter into an unspecific pore [7]. Another member of the mitochondrial carrier family, which has a strong propensity to react with heavy metals, is the carnitine acylcarnitine carrier (CAC, SLC25A20) [8]. This carrier is essential for the β-oxidation pathway since it catalyzes the transport of acyl groups as acylcarnitines into the mitochondrial matrix, where the acyl moieties are processed by the enzymes of the β-oxidation pathway [9]. The transporter works by an antiport mode in which the acylcarnitine substrates are transported into the matrix in exchange for free carnitine. The transporter shows highest affinity for palmitoylcarnitine. It is regulated by several physiological compounds, such as reduced glutathione (GSH), H_2_S and NO [10,11,12] that modulate the transport activity acting on one or two specific Cys residues. The Cys targets have been identified by site-directed mutagenesis, namely C136 and/or C155. The CAC also behaves as an off-target site for drugs [13,14] and mercury compounds, as well. It was recently demonstrated that this transporter is a novel target for mercurial toxicity, since it interacts with mercury compounds at nanomolar concentrations, leading to alteration of locomotion in the animal zebrafish model [8]. Therefore, we investigated the sensitivity of the CAC to copper that has the double face of a physiological cofactor as well as a toxic compound depending on its concentration in cells. The molecular mechanism of the observed effects was revealed.

## 2. Results

Several physiological and non-physiological sulfhydryl reagents have been found to efficiently interact with the CAC causing inhibition or stimulation of the transport function, also depending on the initial redox state of the transporter [11]. Copper, as a divalent (Cu^2+^) or a monovalent (Cu^+^) ion, was tested in this work for its ability to interact with the CAC and to affect the transport function. 

### 2.1. Effect of Copper on the Native CAC and on Intact Mitochondria 

**Figure 1:**
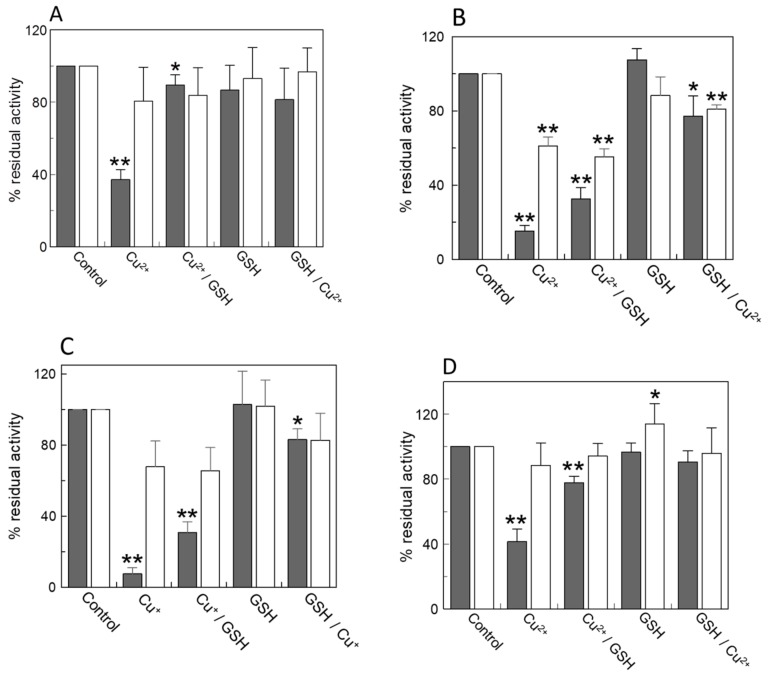
Effect of copper and reduced glutathione (GSH) on native and recombinant carnitine/acylcarnitine carrier (CAC). (**A**) Rat liver mitochondria (5 mg/100 μL) were incubated or not (control) for 1 h, with CuCl_2_ (50 µM) or GSH (5 mM) as indicated. After washing, mitochondria (0.3 mg) were then solubilized with 3% TX-100, 10 mM PIPES pH 7.0 and reconstituted in proteoliposomes for transport assay as indicated in Materials and Methods. (**B**) The CAC was treated with CuCl_2_ or GSH after extraction from mitochondria, as indicated, and then reconstituted in proteoliposomes. Unreacted reagents were removed by gel filtration chromatography (Sephadex G-75) as described in Materials and Methods. (**C**) All the conditions were the same as in (**B**) except that CuCl was used instead of CuCl_2_. (**D**) Purified crude rat liver mitochondria (5 mg/100 μL) were incubated for 2 h with 20 mM carnitine and washed three times with a buffer containing 150 mM sucrose, 50 mM TRIS-HCl and 50 mM KCl. After treatment for 1 h with CuCl_2_ (50 µM) or GSH (5 mM) the transport activity of CAC on intact mitochondria was measured by adding 0.05 mM [^3^H]-carnitine. The uptake was stopped at 30’ by three washes with cold 1 mM NEM added to the same buffer used before. In all panels, black bars and white bars indicate without or with the addition of 5 mM dithioerythritol (DTE) 1 min before the transport assay to aliquots of each sample. The data are expressed as a percent of the controls (without any addition) and are means ± SD from three independent experiments; significantly different from the respective control as estimated by Student’s *t* test (* *p* < 0.05, ** *p* < 0.01).

For testing the effect of copper on the native CAC, intact rat liver mitochondria were incubated with Cu^2+^ in the form of CuCl_2_ (Figure 1). Then, aliquots of treated mitochondria were added with GSH to test the ability of the reducing agent to reverse the possible effects of copper under conditions mimicking the physiological environment. After removal of the unreacted compounds, mitochondria were solubilized with detergents, and the extracted CAC was reconstituted in proteoliposomes for transport assay as [^3^H]carnitine/carnitine antiport. As shown in Figure 1A, the treatment of mitochondria with Cu^2+^ led to a strong inhibition of transport with respect to the protein extracted from untreated mitochondria. The addition of GSH after Cu^2+^ led to a substantial recovery of the transport activity, i.e., the physiological reducing agent was able to reverse the Cu^2+^–protein interaction, indicating that it probably occurred via thiol residues of the protein. As a control, transport was measured in the presence of the sole GSH that did not exert any appreciable effect on the transport activity. The addition of GSH prior to the incubation with Cu^2+^ prevented the inhibition by the heavy metal. To confirm that the inhibition by Cu^2+^ was mediated by interaction with Cys residues of the protein, the SH reducing agent dithioerythritol (DTE) was added to a fraction of all the samples before starting transport (Figure 1A). As expected, DTE reversed the inhibition in all the samples, according to previous findings [8]. Similar results were obtained incubating the CAC with Cu^2+^ after the extraction from mitochondria (Figure 1B). In this case, a stronger inhibition and a lower recovery of the transport activity by GSH were observed. Since it is known that copper can also exist as Cu^+^ in cells, we have tested the monovalent cation in some experiments. The effect of treating the protein with Cu^+^ (Figure 1C), was virtually identical to that of the divalent cation. Both chemical and physiological SH reducing agents reverse the action of monovalent copper, as well. Very similar results were obtained by performing the entire experiment on intact mitochondria (Figure 1D). In this approach, the CAC was not extracted from the mitochondrial membrane but assayed in its cellular location. This approach confirmed the action of copper also in vivo conditions.

**Figure 2:**
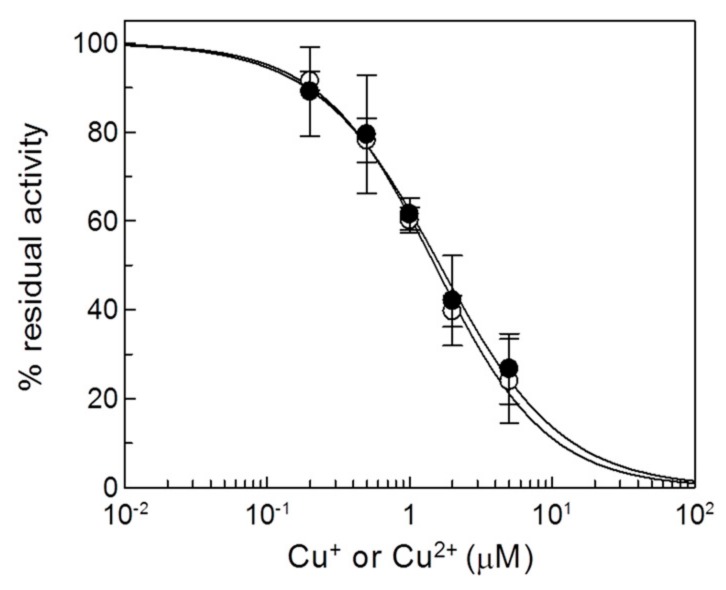
Dose-response analysis of copper effect on the native CAC. Proteoliposomes reconstituted with the CAC extracted from rat liver mitochondria were incubated with (○) CuCl_2_ or (●) CuCl at the indicated concentrations for 1 min, and then the transport activity was measured by adding 0.1 mM [^3^H]carnitine and stopped after 10 min as described in Materials and Methods. Percent of residual activity with respect to the control, without copper treatment, is reported. The values are the means ± SD from three independent experiments.

To gain information on the affinity of the CAC towards the copper ions, a dose-response experiment was performed by measuring the residual transport activity of the CAC extracted from mitochondria, after incubation with different concentrations of Cu^2+^ or Cu^+^ (Figure 2). The data indicated that both the copper forms inhibited the CAC and that the affinity of the protein for the ions was similar, with a calculated IC_50_ of 1.6 ± 0.14 µM or 1.5 ± 0.26 µM for Cu^2+^ or Cu^+^, respectively. 

### 2.2. Effects of Copper on the Recombinant CAC

**Figure 3:**
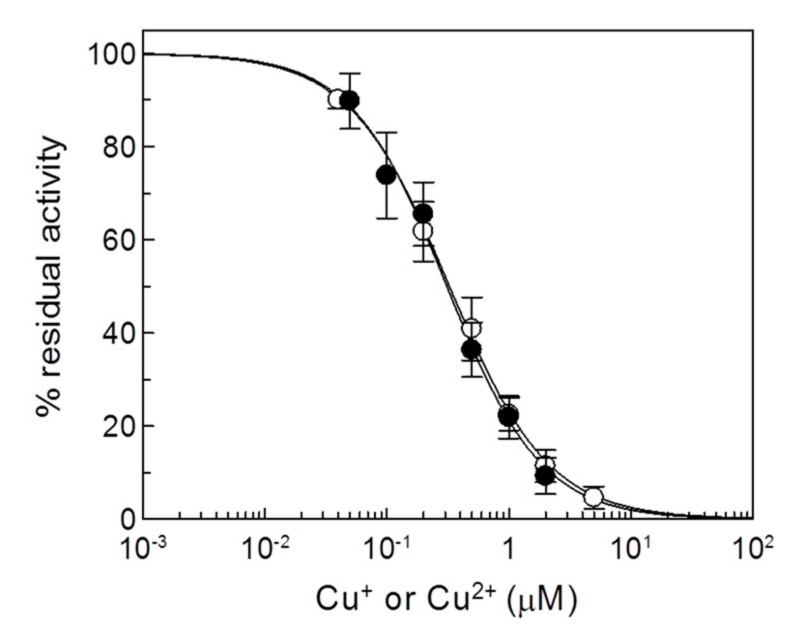
Dose-response analysis of copper effect on the recombinant CAC. Proteoliposomes reconstituted with the recombinant WT CAC were incubated with (○) CuCl_2_ or (●) CuCl at the indicated concentrations for 1 min, and then the transport activity was measured by adding 0.1 mM [^3^H]carnitine and stopped after 10 min as described in Materials and Methods. Percent of residual activity with respect to the control, without copper treatment, is reported. The values are the means ± SD from three independent experiments.

To investigate the mechanism of interaction of copper with the CAC and to ascertain the involvement of specific Cys residues in the mechanism of inhibition of the transport function, the recombinant wild type (WT) protein was employed, together with site-directed Cys mutants. The substitutions of Cys residues of rat CAC resulted in active proteins with a variable but reproducible transport activity, indicating that none of the Cys residues was essential for function and, hence, not directly involved in substrate binding and/or translocation [15] (see also Supplementary Figure S1). Thus, the mutants could be used for obtaining information on the mechanism of interaction of copper with the protein. Transport activities were measured as [^3^H]carnitine/carnitine antiport in proteoliposomes, as for the native protein. Dose-response analysis of inhibition by copper of the WT CAC was firstly performed (Figure 3). The recombinant protein was strongly inhibited by the metal ions with a calculated IC_50_ of 0.28 ± 0.088 or 0.33 ± 0.0061 µM for Cu^2+^ or Cu^+^, respectively. 

**Figure 4:**
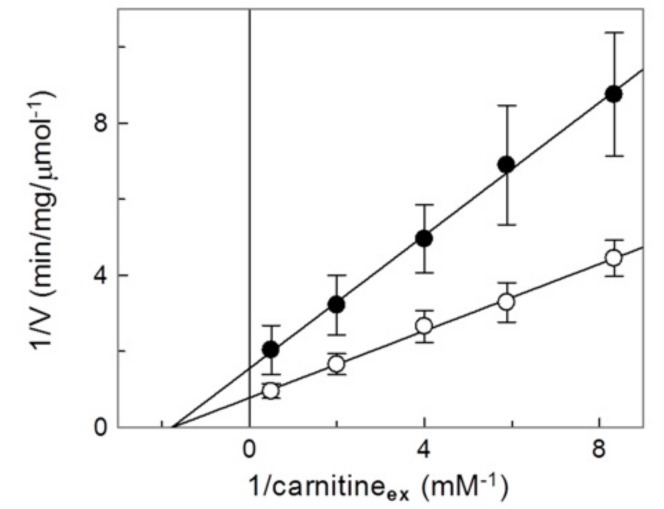
Kinetic analysis of the inhibition by Cu^2+^ of the recombinant CAC. The carnitine/carnitine antiport rate was measured, as described in Materials and Methods, adding [^3^H]carnitine at the indicated concentrations to proteoliposomes containing 15 mM internal carnitine in the absence (○) or in the presence of 1 μM CuCl_2_ added together with the labelled substrate (●). The transport was stopped after 10 min (i.e., within the initial linear range of the time course, see Materials and Methods) by 1.5 mM NEM. Experimental data are plotted according to Lineweaver–Burk as reciprocal transport rate vs. reciprocal carnitine concentrations. Values are means ± S.D. from three independent experiments.

The inhibition of the recombinant proteins by metals was stronger with respect to the native protein, i.e., the IC_50_ was about five times lower. This may be due to both the purity of the recombinant protein, i.e., absence of compounds that prevents copper binding to the CAC (see also Section 2.1). Therefore, the most plausible actual IC_50_ value was the one obtained with the recombinant purified protein. To investigate the influence of the copper interaction with the substrate (carnitine) binding, inhibition kinetics were performed (Figure 4).

**Figure 5:**
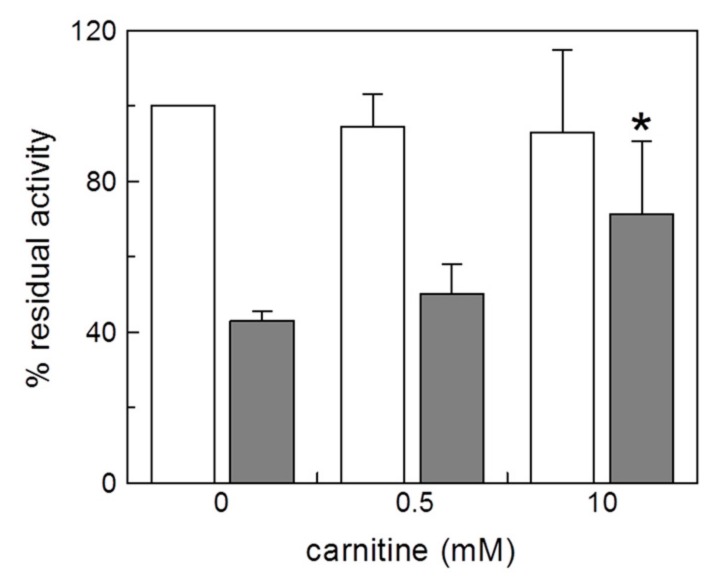
Protection by substrate of the CAC inhibition by Cu^2+^. Carnitine at the indicated concentrations was added to proteoliposomes, and then the mixture was incubated for 1 min with (grey bars) or without (white bars) the addition of 0.5 μM CuCl_2_. Unreacted compound was removed by passing the proteoliposomes through Sephadex G-75 columns (see Materials and Methods). Transport was then started by adding 0.1 mM [^3^H]carnitine to the proteoliposomes and stopped after 10 min. Percent residual activity is reported with respect to the control without the addition of CuCl_2_. The data are the means ± SD from three different experiments; significantly different from the respective control as estimated by the Student’s *t* test (* *p* < 0.05).

From a double reciprocal plot (Lineweaver–Burk) analysis of the experimental data, a variation of Vmax but not of the Km was observed, indicating a non competitive type of interaction of the Cu^2+^ with the transporter. The Ki value derived from the plot was 0.74 ± 0.22 µM. This value was slightly higher than that of the IC_50_ for Cu^2+^, indicating that the inhibition could not be purely non-competitive. Indeed, in case of covalent inhibition due to the strong metal-S bond of copper with Cys residue(s) of the CAC, an apparent non competitive behavior could be observed also if the inhibitor interacted with the substrate binding pocket. To ascertain whether the substrate may, anyway, interfere with the binding of Cu^2+^, a protection experiment was performed in which carnitine was added to the CAC at different concentrations, before the inhibitor (Figure 5). As shown in the figure, the addition of carnitine partially prevented the inhibition, indicating that the target of copper ion could be a Cys residue that was close to the substrate binding site or within the substrate transport path. Controls were also performed in which carnitine was added in the absence of Cu^2+^ to exclude effects on the transport activity independent of the addition of the metal; in this case no variations were observed (Figure 5). This data suggested the involvement of C136 and also C155, which are in or are related to the transport path of CAC [16]. 

**Figure 6:**
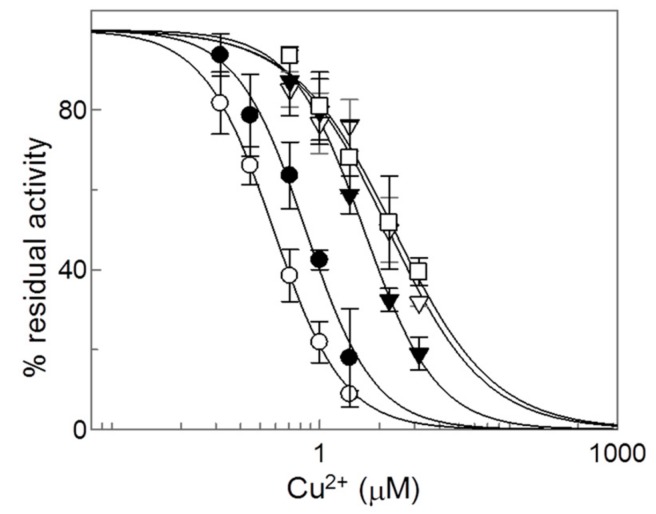
Dose-response analysis of copper effect on Cys mutants of CAC. (○) WT, (●) C23S/C58S/C89S/C283S, (▽) C136S, (▼) C155S and (□) C136S/C155S. After 1 min of incubation with Cu^2+^ at the indicated concentrations, transport activity was measured by adding 0.1 mM [^3^H]-carnitine and stopped after 30 min as described in Materials and Methods. Percent of residual activity with respect to the control, without Cu^2+^ treatment, was reported. The values were the means ± SD from three experiments.

In order to identify the residue(s) actually involved in the Cu^2+^ interaction and inhibition, the site-directed Cys mutants were tested for inhibition (Figure 6).

The sensitivity to Cu^2+^ was strongly decreased in the mutants C136S and C155S, indicating that the two Cys residues played an important role in the interaction. The IC_50_ was indeed about one order of magnitude higher than that of the WT. To confirm the involvement of the two residues, the double mutant C136/155S was tested for inhibition. Additionally, in this case, a ten times higher IC_50_ value was found. As further positive proof for the crucial role of the two Cys residues, a mutant containing only C136 and C155 (C23S/C58S/C89S/C283S) was tested. In this case, a strong inhibition was again observed, even though the IC_50_ value was somewhat higher than in the WT. The data indicate that the inhibition of the CAC was not due to oxidation of thiol groups of the C136 and C155 by Cu^2+^ but by interaction with two Cys residues, namely C136 and C155 forming a sulfur–metal bond. The substitution of the crucial Cys residues with Ser, indeed, decreased the strength of the bonds. The finding that the increase of IC_50_ was maximal upon substitution of both the critical Cys residues lets us hypothesize that the copper ion may form a cross-link with the two Cys residues.

### 2.3. Computational Analysis of the Interaction of Copper with the CAC

**Figure 7:**
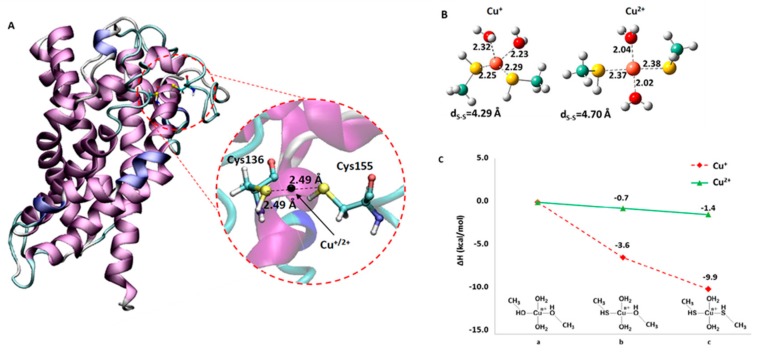
Computational analysis of the CAC–copper interaction. (**A**) Molecular dynamics frame (at 150 ns) of WT CAC revealing the minimum distance between C136 and C155 as evidenced in red circled region. The middle point indicates the position for copper ions. The orientation of the CAC is matrix side (up), cytosolic side (down). (**B**) B3LYP-D3/6-31+G(d,p) optimized geometries for the smaller models of (Cu^+^(CH_3_S(H))_2_(H_2_O)_2_) and (Cu^2^^+^–(CH_3_S(H))_2_(H_2_O)_2_). Main geometrical parameters are reported in Å. (**C**) Effect of the gradual substitution of cysteine by serine (from right to left in the graph) on the binding energy (ΔH) for the Cu^2+^ and Cu^+^ complexes.

To gain further insights into the formation of Cu^2+^ or Cu+ cross-link with thiol groups of Cys residues, a computational approach was employed. In fact, the conformational behavior of apo-protein WT CAC (Figure 7) was analyzed for all the 200 ns of productive molecular dynamics simulation (MDs). In particular, a careful examination was devoted to the orientation of all the cysteine residues present (C23, C58, C89, C136, C155 and C283) for WT-CAC and also for its C136S and C155S CAC mutated forms. Results reported in Supplementary Figures S2–S4 show the C136 and C155 residues lie at an average distance between the sulphur atoms (S–S) equal to 10.0 Å with a minimum of 4.98 Å (Figure 7A). This distance value is coherent with possible coordination of Cu^2+^/Cu^+^ metal ion by the sulphur atom of cysteine, as reported in the literature for other proteins [17,18]. With the aim to obtain atomistic insights about this aspect, smaller model systems (Cu^+^–(CH_3_S(H))_2_(H_2_O)_2_) and (Cu^2+^–(CH3S(H))_2_(H2O)_2_), depicted in Figure 7B, were undertaken to carry out an investigation at density functional theory (DFT) level. The optimized coordination distances well depicted what was observed during the MDs. Further details on the Cu^2+^ and Cu^+^ ions binding affinity of the thiol group were given by checking the effect on the energy due to the gradual substitution of -SH by -OH simulating the serine side chain (Figure 7C and for the corresponding optimized species see Supplementary Figure S5). The ΔH became higher (from right to the left of Figure 7C) when the thiol groups were replaced by hydroxyl ones, suggesting a better affinity of the copper ions for -SH group in accordance with what the hard–soft acid–base theory proposes. The computational data correlated well with the data obtained with the site-directed mutants and were in favor of the formation of two S–Cu^2+/+^ bonds with the thiol groups of C136 and C155. By assuming that the used models (**a**, **b** and **c** of Figure 7C) are representative of the apo protein, their relative deprotonation energies reflected the effect of the Cu^2+^/Cu^+^ binding on the acidity of the coordinated thiol groups (Figure S5) as analogously found in other proteins [19,20]. All the experimental and the computational data concurred in explaining the mechanism of inhibition and correlated well with previous data showing that C136 and C155 are involved in conformational changes required for the transport process [10,11,12,16,21,22,23,24]. 

### 2.4. Analysis of the Inhibition by Copper on the C-Less CAC

**Figure 8:**
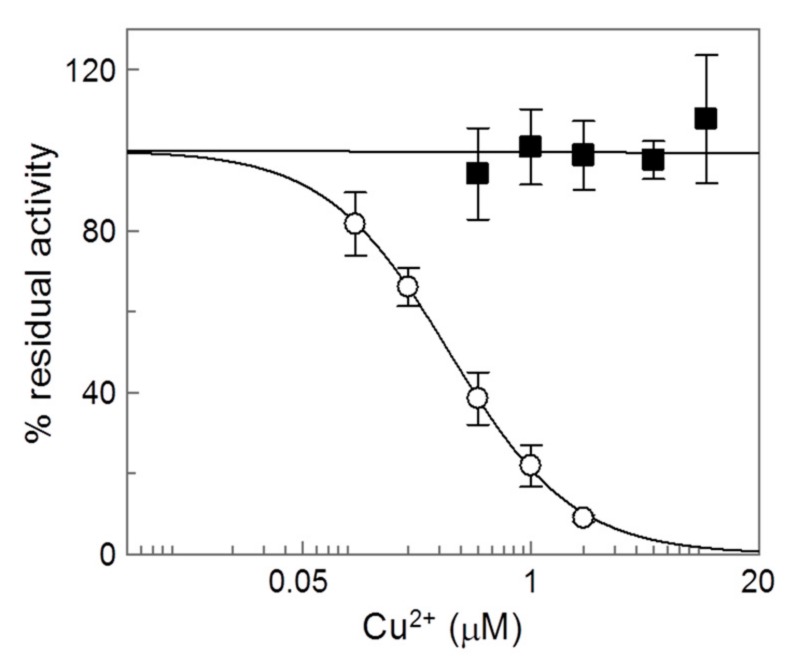
Dose-response analysis of copper effect on the C-less mutant of CAC. (○) WT and (■) C23V/C58V/C89S/C136V/C155V/C283S. After 1 min of incubation with Cu^2+^ at the indicated concentrations, transport activity was measured by adding 0.1 mM [^3^H]-carnitine and stopped after 30 min as described in Materials and Methods. Percent of residual activity with respect to the control, without Cu^2+^ treatment, was reported. The values were the means ± SD from three experiments.

As final proof of the involvement of Cys residues in the interaction with copper, the C-less CAC was tested (Figure 8). To obtain a functional C-less, most of the Cys residues had to be substituted by Val (C23V/C58V/C89S/C136V/C155V/C283S) [22]. As shown in Figure 8, this mutant was completely insensitive to the metal. This finding finally demonstrated the involvement of Cys residues in the interaction. Thus, this data confirmed the essential requirement for metal-S bonds in the interaction with C136 and C155. Moreover, it confirmed also that if some interactions still occur with the OH of C89 and C283 (which are Ser residues in the C-less), these residues are not crucial for inhibition. Indeed, C89 or C283 were located in the upper part of the large water filled cavity where the steric hindrance of the relatively small copper ions bound to the Cys residues did not prevent transport by the metal ion binding. Moreover, in this location the residues could not come close enough to allow a copper bridge with both residues. Thus, as opposed to the binding of large size molecules such as omeprazole that cause inhibition, as previously described [14], a hypothetical binding of Cu^2+^ to these residues did not impair the transport activity.

## 3. Discussion

The reported data clearly show that copper interacts with the CAC inhibiting the transport function in a concentration dependent manner. The effect of Cu^2+^ or Cu^+^, tested in some of the experiments, appears very similar, indicating that the inhibition of the CAC is not influenced by the oxidation state of the cation and/or that it is not due to a redox action of copper on the thiol groups of the protein. The interaction with the CAC occurs mainly with two Cys residues, namely C136 and C155, that form a sulphur–metal bond as confirmed by the computational analysis. The Cu^2+^ or Cu^+^ ions can form two bonds with the facing amino acid residues whose energies decrease by substituting SH (Cys) with OH (Ser) (see Figure 7). The increase in IC_50_ upon substitution of one of the two residues is very similar, indicating that either Cys residues are involved in the interaction. This indicates that Cu^2+^ or Cu+ binds to both Cys residues by bridging with the two thiols for optimal interaction (lowest IC_50_), correlating well with the computational analysis. The much higher IC_50_ exhibited by the double mutant C136/155S confirms this hypothesis. C136 and C155 must be in a reduced state for full activity of the protein, since these residues move with respect to each other during the translocation process [16]. In the cytosolic opened conformation (c-state), the residues are closer than in the matrix opened conformation (m-state), due to the changing location of the second (h3–4) large matrix hydrophilic loop [23]. The oxidized (disulphide) state of the two Cys residues prevents the conformational changes needed for translocation [16]. Binding of Cu^2+^ to the two Cys residues has the same inhibitory effect of disulphide (Figure 7C). For all the other Cys residues, only a single S–metal bond could be possible due to the larger distance among the thiol residues (Supplementary Figure S2). Thus, a lower affinity binding of copper will lead to a higher IC_50_ with respect to the binding with C136/C155. This is evident in the mutants lacking both C136 and C155. We cannot exclude that some Cys residues different from C136 or C155 could bind Cu^2+^ with a relatively high affinity, but without any consequences on the transport activity. As an example, C89 or C283, which are located in the large water filled cavity of the CAC, might be targeted by copper. However, in the large upper part of the cavity, the steric hindrance of the relatively small Cu^2+^ ion bound to the Cys residues does not prevent transport, differently from the binding of large size molecules such as omeprazole that cause inhibition, as previously described [14]. The interaction of copper with other Cys residues, which, however, are not relevant for the CAC transport function, will be investigated in future studies, performed by mass spectrometry, for a final conclusion.

Interestingly, both chemical and physiological SH reducing agents reverse the action of copper. On these bases and according to the concentrations of GSH that reverse the inhibition, we may hypothesize that under normal physiological redox conditions, i.e., a relatively high GSH/GSSG ratio [10], copper should be, at least partially, scavenged by GSH. This is in line with the lower inhibition observed after treatment of intact mitochondria with respect to that observed on the isolated protein or on the recombinant protein. Indeed, in a condition closer to the in vivo environment (intact mitochondria) the redox potential of the intermembrane space could be influenced by the presence of GSH or other redox systems, which are very active in this location [10]. To exert some effects on CAC, the concentration of copper in the intermembrane space should reach levels around the IC_50_ for CAC. It is known that the copper level in cells is around 15–40 µM. The free copper concentration does not exceed 5% of the total ([25] and refs herein). According to the current knowledge, copper is transported into the mitochondrial matrix and then is transferred to the membrane complexes [2]. The transport process is mediated by the PIC transporter [5] whose Km for Cu^2+^ is 15 µM. According to this finding, it is very plausible that the concentration of free copper in the intermembrane space could reach values higher than the basic concentration (0.5–2 µM), to be transported into the matrix by the PIC. Thus, it is plausible that, in vivo, Cu^2+^ may interact with the CAC, which faces towards the intermembrane space, during the process of transport mediated by the PIC. However, few or no information is available about the actual concentration of copper and its possible oscillations into the intermembrane space. 

Under several pathological conditions, the copper homeostasis can be altered, and the local free copper concentration can vary. It is known that an increased level of free copper leads to production of ROS [25]. The action of the increased copper concentration on CAC will slow down the β-oxidation flux and hence the production of reducing equivalents. This action will, in turn, slow down the production of ROS by the respiratory chain. 

Other specific pathological states lead to strong increase of copper levels, up to ten times the normal level. This is the case of Wilson’s disease, caused by a failure in copper excretion; similar alterations could occur in ALS [25]. The strongly increased copper concentration will cause inhibition of the CAC, resulting in a durable impairment of the β-oxidation of fatty acids. This may contribute at the molecular level to the mitochondrial dysfunction occurring in this pathology. The described data have a toxicological relevance too, under environmental conditions in which the level of heavy metals, including copper, increases. Under such a condition, an impairment of the CAC function may cause a decrease of the mitochondrial β-oxidation flux with alterations of the energy metabolism.

## 4. Materials and Methods 

### 4.1. Materials

L-[methyl-^3^H]carnitine from Scopus Research BV Costerweg, Sephadex G-75, egg-yolk phospholipids (l-α phosphatidylcholine from fresh turkey egg yolk), PIPES, Triton X-100, cardiolipin and L-carnitine were purchased from Sigma-Aldrich, Milan, Italy. All other reagents were of analytical grade.

### 4.2. Overexpression of the WT and Mutant CACs 

The pMW7-WTratCAC recombinant plasmid was used for over-expressing the rat CAC, as previously described [26]. To introduce the mutations the overlap extension method was used as described introducing the NdeI and HindIII restriction sites [27]. The PCR products were purified by the QIAEX II Gel Extraction Kit (QIAGEN) and digested with NdeI and HindIII and then ligated into the pMW7. All mutations were verified by DNA sequencing. The resulting plasmid constructs were transformed into *E. coli* C0214. Overexpression, inclusion body fraction preparation and purification of the CAC proteins were performed as previously described [28].

### 4.3. Reconstitution of CAC in Proteoliposomes 

The recombinant CAC (WT and mutant) proteins were used for proteoliposome formation as previously described [28]. For the experiments with the native protein, rat liver mitochondria were first purified using the standard procedure of cell disruption and differential centrifugation [29]. Protein was extracted with 3% Triton X-100 and used for proteoliposome formation as above described for the recombinant protein, in the absence of reducing agents. The concentration of intraliposomal carnitine was 15 mM in all samples. 

### 4.4. Transport Assay in Proteoliposomes

For transport assay in proteoliposomes, the external substrate was removed from proteoliposomes by passing 550 μL of proteoliposomes through a Sephadex G-75 column. Then, 600 μL of the turbid eluate were collected, divided into samples of 100 μL each and used for transport measurement by the inhibitor–stop method [30]. 

Transport was started by adding 0.1 mM [^3^H]carnitine to proteoliposome samples and, at the indicated time interval stopped by 1.5 mM NEM. NEM was added together with the labelled substrate at time zero in the control samples. After terminating the transport reaction, the external substrate was removed by chromatography on Sephadex G-75 columns, and the intraliposomal radioactivity was measured [30]. Transport was measured at 25 °C. The experimental values were corrected by subtracting the controls. Transport rates were measured in 5 min, i.e., within the initial linear range of the time course. 

### 4.5. Computational Methods

The starting structure of CAC protein was obtained from homology modeling simulation. Two additional mutated structures were obtained by the in silico mutation C136S and C155S, respectively. The protonation state for each titrable residue was assigned using the H++ web server. The three different proteins were respectively surrounded by a rectangular box (62 × 61 × 68 Å^3^) that was filled with water molecules, within 12 Å from the surface of the protein, and 13 Cl-counter-ions. The entire systems were minimized and successively undertaken to MDs adopting AMBER16 software package, [31] following the procedure previously applied [32]. 

DFT calculations were carried out using B3LYP functional as implemented in Gaussian09 D.01 software [33] and taking in to account the adequate electronical treatment of each atom (H, C, N, O, S and Cu) and the effect of protein surrounding, according to accurate protocol successfully adopted in similar works, [19,20,34]. All geometries were optimized, and frequency calculations were performed to confirm their nature of minima. Total interaction energies (ΔH interaction) were obtained according to the counterpoise method, as a sum of two body interaction energies, [35] for the following reaction:

Cu^n+^ + aCys + bSer + 2H_2_O → (Cu(Cys)_a_(Ser)_b_(H_2_O)_2_)^n+^

*n* = 1, 2 a, b = 0, 1, 2

### 4.6. Other Methods

The amount of reconstituted protein was estimated from Coomassie blue stained SDS–PAGE gels by using the Chemidoc imaging system equipped with Quantity One software (Bio-Rad) as previously described [36].

## 5. Conclusions

We demonstrate that copper interacts with the CAC and strongly inhibits its function. The molecular mechanism of the interaction was revealed by combining the transport assay of site-directed mutants with computational chemistry. All the data concur in demonstrating the occurrence of a cross-link among copper and two Cys residues of the CAC that are crucial for the conformational changes underlying the transport process. From a pathophysiological point of view, on the one hand, the functional inhibition may modulate the CAC and hence the β-oxidation contribution in protecting the cell from ROS that are produced during temporary alterations of the copper homeostasis [25]. On the other hand, under specific pathological states such as Wilson’s disease, in which the copper homeostasis is irreversibly altered [25], the effect of the heavy metal on the CAC would result in a strong impairment of fatty acid utilization, worsening the energy metabolism.

## Supplementary Materials

The following are available online. Figure S1: Interatomic average distances, calculated along 200 ns of MDs for the three different systems, between residues deputed to bind the copper ions, Figure S2: Root Mean Square Deviation (RMSD) calculated along 200 ns of MDs for the three different systems, Figure S3: Root Mean Square Fluctuation (RMSF) calculated along 200 ns of MDs for all the amino, acid residues of the three systems, Figure S4: B3LYP-D3/6-31+G(d,p) optimized structures of [Cu^+^(CH_3_SH)(CH_3_OH)(H_2_O)_2_], [Cu^+^(CH_3_OH)_2_(H_2_O)_2_], [Cu^++^(CH_3_SH)(CH_3_OH)(H_2_O)_2_], and [Cu^++^(CH_3_OH)_2_(H_2_O)_2_], Figure S5: Calculated ΔHdeprotonation for [Cu^n+^(CH_3_SH)_2_(H_2_O)_2_] → [Cu^n+^(CH_3_S)_2_(H_2_O)_2_]^n-2^ + 2H^+^; [Cu^n+^(CH_3_SH)(CH_3_OH)(H_2_O)_2_] → [Cu^+^(CH_3_S)(CH_3_O)(H_2_O)_2_]^n-2^ + 2H^+^ and [Cu^n+^(CH_3_OH)_2_(H_2_O)_2_] → [Cu^n+^(CH_3_O)_2_(H_2_O)_2_]^n-2^ + 2H^+^.
