# Supplementary material for: Effect of Copper on the Mitochondrial Carnitine/Acylcarnitine Carrier Via Interaction with Cys136 and Cys155. Possible Implications in Pathophysiology
Source: Molecules. 2020 Feb 13;25(4):820. doi: 10.3390/molecules25040820 (PMC7070283; doi:10.3390/molecules25040820)
Supplement: Supplementary file 1 [file molecules-25-00820-s001.pdf]

Effect of copper on the carnitine transport mediated by the mitochondrial carnitine/acylcarnitine carrier. Structural basis and possible implications in pathophysiology.

Nicola Giangregorio[1a], Annamaria Tonazzi[1a], Lara Console[2], Mario Prejanò[3], Tiziana Marino[3], Nino Russo[3] and Cesare Indiveri[2,*]

[1] CNR Institute of Biomembranes, Bioenergetics and Molecular Biotechnologies (IBIOM) via Amendola 122/O, 70126 Bari, Italy

[2] Department DiBEST (Biologia, Ecologia, Scienze della Terra) Unit of Biochemistry and Molecular Biotechnology, University of Calabria, Via Bucci 4C, 87036 Arcavacata di Rende, Italy

[3] Department CTC (Chemistry and Chemical Technology) University of Calabria, Via Bucci 14C, 87036 Arcavacata di Rende, Italy

[a] These authors contributed equally to this work.

*Correspondence: E-mail: cesare.indiveri@unical.it; Tel:+39-0984-492939; (C.I.)

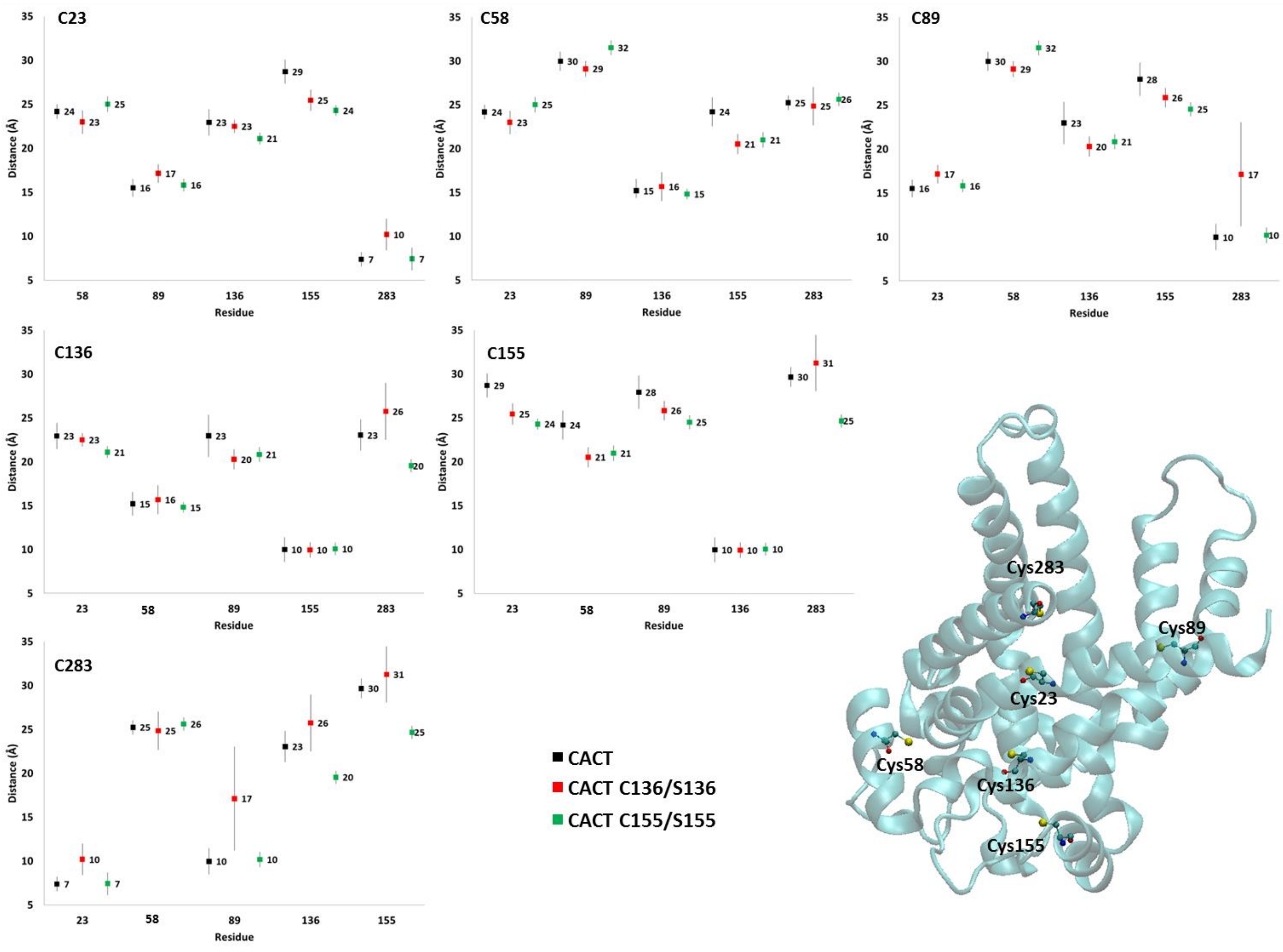

Figure S1. Interatomic average distances, calculated along 200 ns of MDs for the three different systems, between residues deputed to bind the copper ions. The reported standard deviation bar were evaluated according to the equation $\sigma = \sqrt{\frac{\sum_{i=1}^{N}\left(d_i - \bar{d}\right)^2}{N-1}}$.

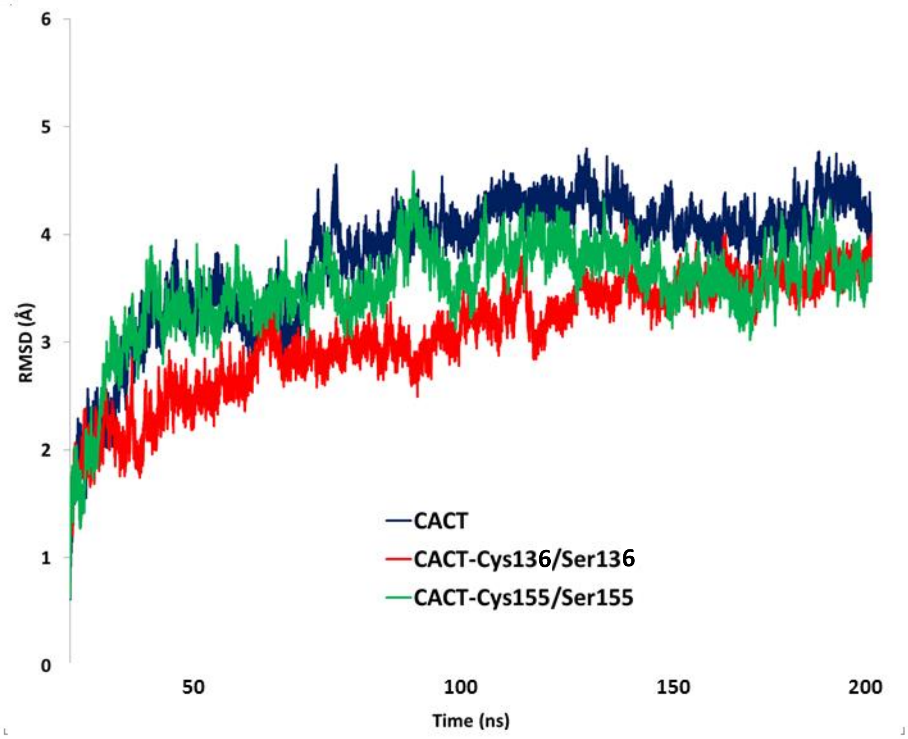

Figure S2. Root Mean Square Deviation (RMSD) calculated along 200 ns of MDs for the three different systems.

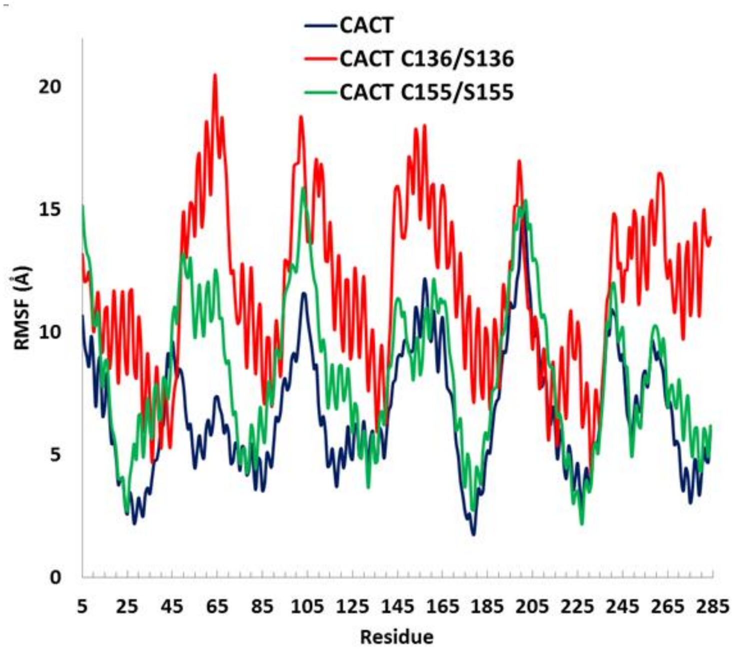

Figure S3. Root Mean Square Fluctuation (RMSF) calculated along 200 ns of MDs for all the amino acid residues of the three systems.

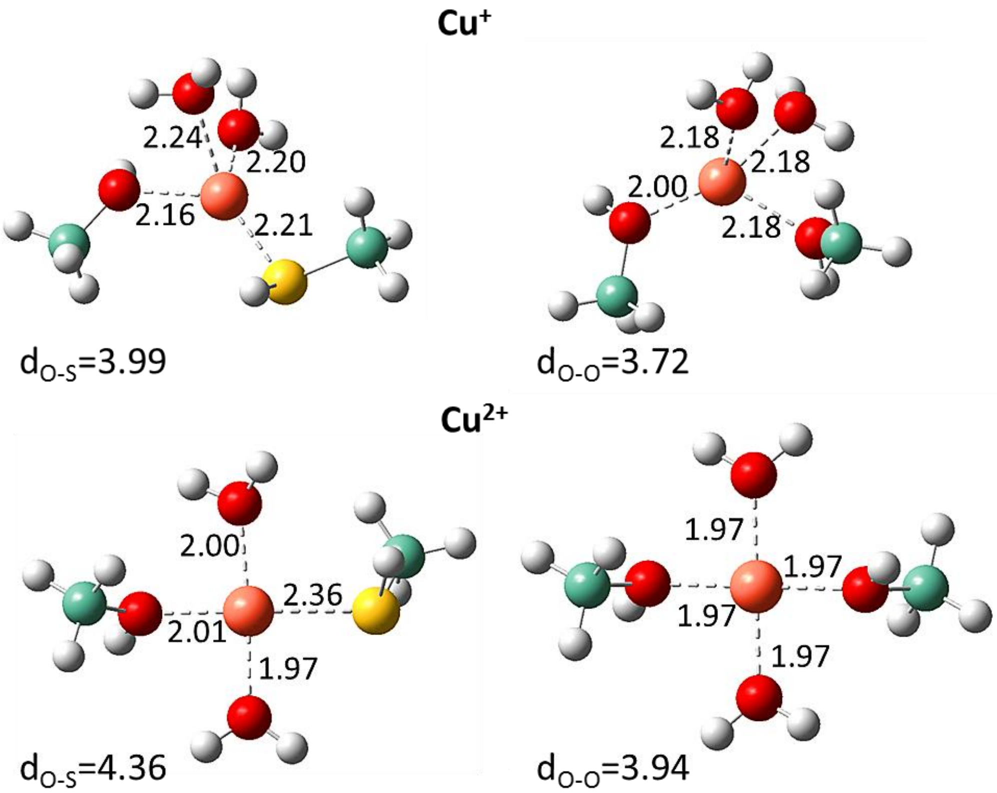

Figure S4. B3LYP-D3/6-31+G(d,p) optimized structures of $[Cu^{+}(CH_3SH)(CH_3OH)(H_2O)_2]$, $[Cu^{+}(CH_3OH)_2(H_2O)_2]$, $[Cu^{++}(CH_3SH)(CH_3OH)(H_2O)_2]$, and $[Cu^{++}(CH_3OH)_2(H_2O)_2]$. Main geometrical parameters are reported in (Å).

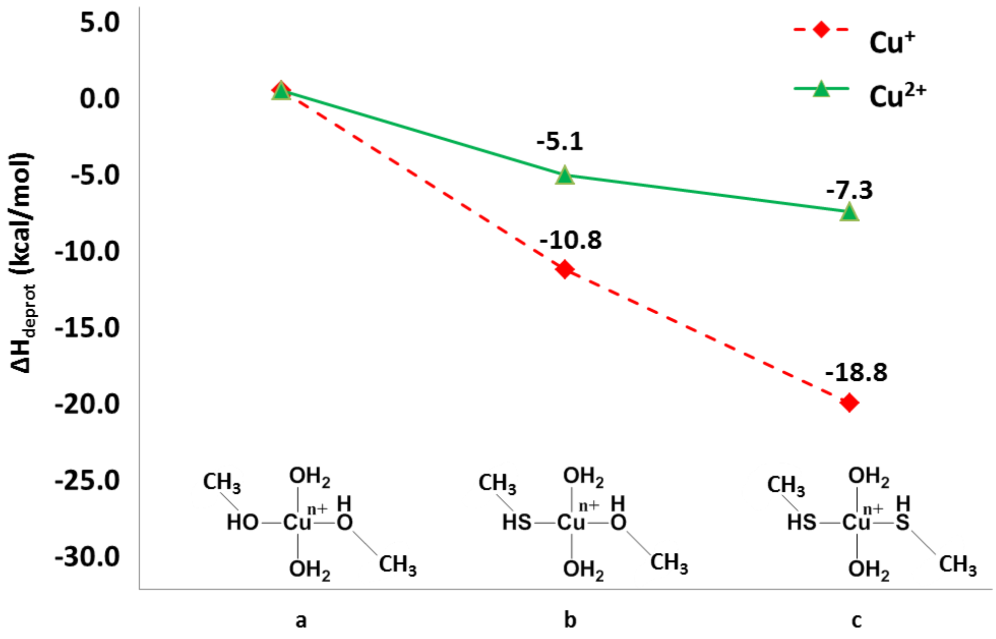

Figure S5. Calculated $\Delta H_{deprotonation}$ according to the following reactions:

a) $[Cu^{n+}(CH_3SH)_2(H_2O)_2] \rightarrow [Cu^{n+}(CH_3S)_2(H_2O)_2]^{n-2} + 2H^+$

b) $[Cu^{n+}(CH_3SH)(CH_3OH)(H_2O)_2] \rightarrow [Cu^{+}(CH_3S)(CH_3O)(H_2O)_2]^{n-2} + 2H^+$

c) $[Cu^{n+}(CH_3OH)_2(H_2O)_2] \rightarrow [Cu^{n+}(CH_3O)_2(H_2O)_2]^{n-2} + 2H^+$
